# Site Dependency of Anodal Transcranial Direct-Current Stimulation on Reaction Time and Transfer of Learning during a Sequential Visual Isometric Pinch Task

**DOI:** 10.3390/brainsci14040408

**Published:** 2024-04-22

**Authors:** Fahimeh Hashemirad, Maryam Zoghi, Paul B. Fitzgerald, Masoumeh Hashemirad, Shapour Jaberzadeh

**Affiliations:** 1 Department of Physical Therapy, University of Social Welfare and Rehabilitation Sciences, Tehran 1985713871, Iran; 2 Monash Neuromodulation Research Unit, Department of Physiotherapy, School of Primary and Allied Health Care, Faculty of Medicine, Nursing and Health Sciences, Monash University, Melbourne, VIC 3199, Australia; shapour.jaberzadeh@monash.edu; 3 Discipline of Physiotherapy, Institute of Health and Wellbeing, Federation University, Ballart, VIC 3199, Australia; m.zoghi@federation.edu.au; 4 School of Medicine and Psychology, Australian National University, Canberra, NSW 2601, Australia; paul.fitzgerald@anu.edu.au; 5 Department Mathematics, Azahra University, Tehran 1993893973, Iran; massi67123@gmail.com

**Keywords:** anodal transcranial direct-current stimulation, a-tDCS, reaction time, transfer learning, primary motor cortex, dorsolateral prefrontal cortex, posterior parietal cortex

## Abstract

Considering the advantages of brain stimulation techniques in detecting the role of different areas of the brain in human sensorimotor behaviors, we used anodal transcranial direct-current stimulation (a-tDCS) over three different brain sites of the frontoparietal cortex (FPC) in healthy participants to elucidate the role of these three brain areas of the FPC on reaction time (RT) during a sequential visual isometric pinch task (SVIPT). We also aimed to assess if the stimulation of these cortical sites affects the transfer of learning during SVIPT. A total of 48 right-handed healthy participants were randomly assigned to one of the four a-tDCS groups: (1) left primary motor cortex (M1), (2) left dorsolateral prefrontal cortex (DLPFC), (3) left posterior parietal cortex (PPC), and (4) sham. A-tDCS (0.3 mA, 20 min) was applied concurrently with the SVIPT, in which the participants precisely controlled their forces to reach seven different target forces from 10 to 40% of the maximum voluntary contraction (MVC) presented on a computer screen with the right dominant hand. Four test blocks were randomly performed at the baseline and 15 min after the intervention, including sequence and random blocks with either hand. Our results showed significant elongations in the ratio of RTs between the M1 and sham groups in the sequence blocks of both the right-trained and left-untrained hands. No significant differences were found between the DLPFC and sham groups and the PPC and sham groups in RT measurements within the SVIPT. Our findings suggest that RT improvement within implicit learning of an SVIPT is not mediated by single-session a-tDCS over M1, DLPFC, or PPC. Further research is needed to understand the optimal characteristics of tDCS and stimulation sites to modulate reaction time in a precision control task such as an SVIPT.

## 1. Introduction

The acquisition of sequence learning is critical for daily life [1]. Improvements in performance with practice and repetition are expressed by significant changes in behavioral outcome measures such as reaction time (RT) or error rate [2]. The sequential knowledge and improvements acquired through practice using one hand transfer to the opposite side, in a phenomenon which is called “intermanual transfer” [3,4,5]. 

A network of cortical and subcortical structures is involved in acquiring motor sequence learning [6,7,8,9] and transfer learning to the untrained hand [10,11,12,13,14]. Neuroplasticity in different areas of the frontoparietal cortex (FPC) such as the primary motor cortex (M1), the dorsolateral prefrontal cortex (DLPFC), or the posterior parietal cortex (PPC) has been reported during motor sequence learning [15,16,17]. Shorter RTs in response to expected visual stimuli have been mainly associated with the increased activation of the PPC [18], which is strongly connected with sensorimotor integration for perception and action [19]. During the process of learning, the DLPFC is activated for the inhibition of unrelated stimuli to produce the best response to stimuli in difficult task demands [20]. The M1 is a key motor area that is mainly activated in the process of acquiring a motor skill through the sustained learning of complex movements [21,22,23,24]. In this study, our objective was to explore the contribution of three distinct brain regions to the enhancement of temporal processing in sequential motor learning and the transfer of this learning to the untrained hand. Although neuroimaging studies reveal important insights into the brain areas involved in motor timing, further research is needed to determine the essential role of different areas of the FPC in the reduction in RTs as one of the most important temporal variables during motor learning.

In light of the benefits of transcranial direct-current stimulation (tDCS) for identifying the contributions of specific brain regions to human sensorimotor behaviors [25,26,27,28,29,30], we employed anodal tDCS (a-tDCS) to explore the most effective stimulation locations for enhancing reaction times (RTs) and facilitating the transfer of this improvement to the untrained hand. Even though there are some studies on the effects of a-tDCS on different areas during a serial reaction time task (SRTT) [31,32,33,34], little is known about the effects of brain stimulation on a sequential visual isometric pinch task (SVIPT), which is a force control sequenced task with greater motor demands compared to SRTTs [35,36,37]. Therefore, in the current study, we aimed to investigate whether a-tDCS over three stimulation sites of the FPC (DLPFC, M1, or PPC) could differentially affect RTs during an SVIPT. We also aimed to explore whether these effects are transferred to the untrained hand. 

## 2. Methods and Materials

### 2.1. Participants and Study Design

**Figure 1:**
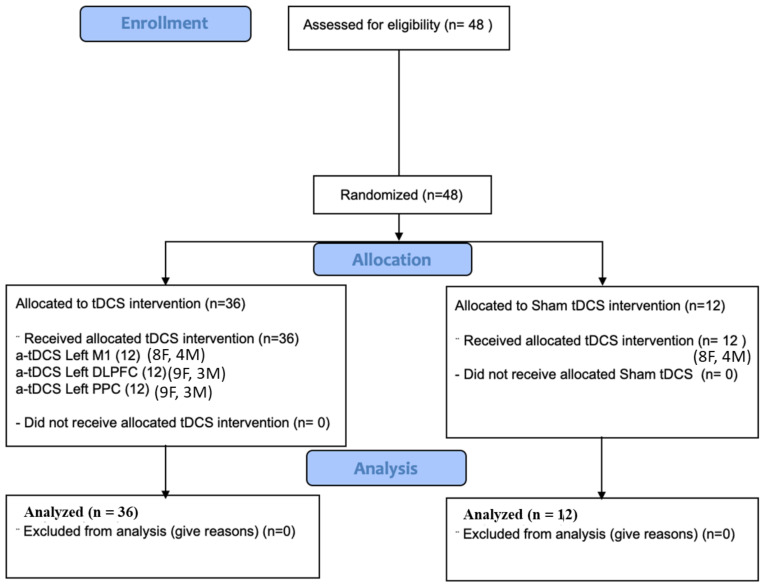
CONSORT flow diagram.

Convenience sampling was employed to recruit the participants in this study, which was a parallel randomized single-blind sham-controlled study. Forty-eight healthy right-handed students (34 females, 14 males; 25.83 ± 6.174) from Monash University participated in this study. For the allocation of the participants in each intervention, a random numbers’ table was used in this study. As gender can influence tDCS outcome [38], the random allocation table was balanced in terms of gender to make sure that males and females were equally distributed among the different groups. The participants were blinded to the experimental conditions while the researcher (FH) administering the tDCS was not blinded. The participants were randomly assigned to one of the four stimulation groups: (1) a-tDCS of left M1, (2) a-tDCS of left DLPFC, (3) a-tDCS of left PPC, and (4) sham a-tDCS (Figure 1). All the participants were right-handed, based on the Edinburgh Handedness Inventory. Information about their experience with computer games was also obtained through a brief questionnaire. Since the participants were all recruited from a pool of Monash University students, the level of educations was similar in all the groups. The participants’ eligibility for tDCS application was determined through a questionnaire comprising inquiries regarding a history of seizures or familial susceptibility, severe headaches, pregnancy, presence of metallic objects in the head, and current medication usage potentially impacting brain function, motor learning, or cognition. Indeed, the exclusion criteria encompassed contraindications to tDCS, a past medical history of neurological or psychiatric disorders, and substantial experience with playing musical instruments. All the participants were naive to the purpose of the experiments. All the participants signed a consent form before taking part in our experiment. This study was approved by the Human Ethics Committee at Monash University, which follows the declaration of Helsinki (F13/3302_2013001720). 

### 2.2. Procedure

**Figure 2:**
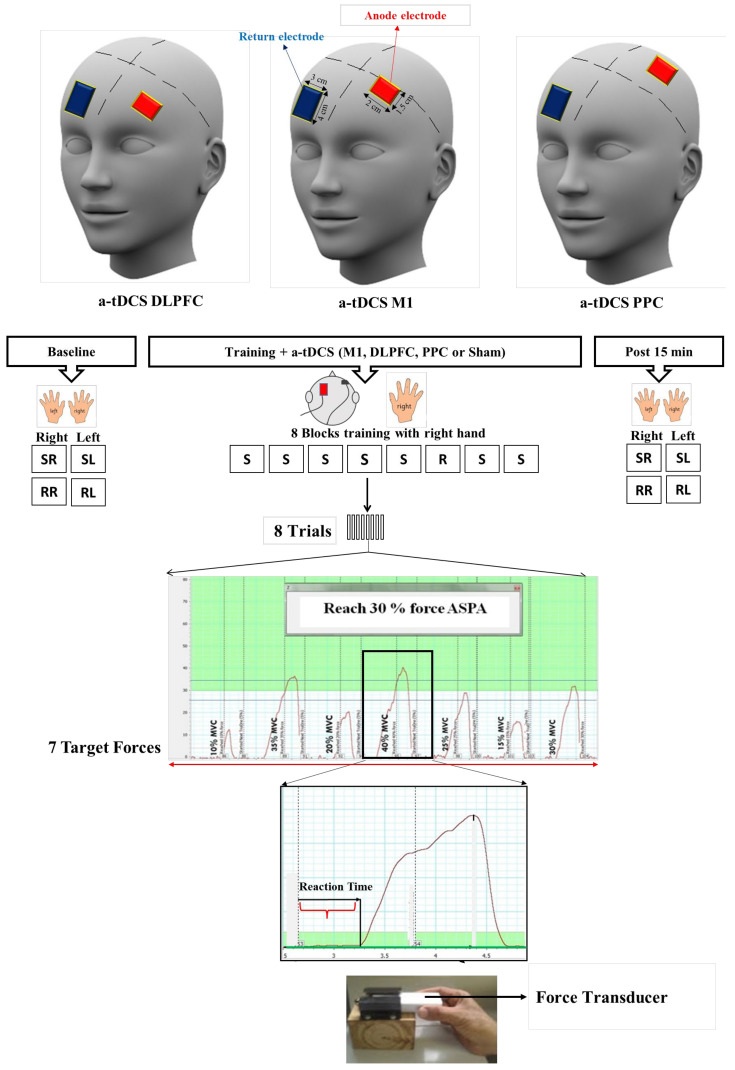
Experimental set up. The participants were instructed to squeeze a force transducer as precisely as possible to reach each target force that appeared on the computer screen. Each sequence block consisted of eight trials, which included seven different target forces from 10 to 40% of their MVC. In a sequence block, the target forces appeared in a sequence order (10, 35, 20, 40, 25, 15, and 30% of the MVC), while the target forces were randomly presented in a random block. They were asked to complete each block as quickly and accurately as possible. The RT was measured as a temporal variable for each target force. SVIPT: sequential visual isometric pinch task; A-tDCS: anodal transcranial direct-current stimulation; M1: primary motor cortex; DLPFC: dorsolateral prefrontal cortex; PPC: posterior parietal cortex; S: sequence block; R: random block; RT: reaction time; SR: sequence right; SL: sequence left; RR: random right; and RL: random left.

A force transducer (AD Instrument MLT004/ST, Bella Vista, NSW, Australia) was used for the SVIPT [39] (Figure 2). For this task, the participants were instructed to squeeze a force transducer between their thumb and index finger and match their force production on the force transducer as precisely and quickly as possible to reach each target force that appeared on the computer screen. PowerLab^TM^ (Bella Vista, NSW, Australia) (4/35) was used and directly connected to the force transducer to convert voltage signals to digital signals. In order to calibrate the force transducer, we determined the maximum isometric contraction (MVC) for each participant individually. This MVC value was then utilized for calibration purposes within the Power Lab data acquisition system, following the methodology outlined in a previous study [39]. The target forces were designed from 10 to 40% of MVC in each trial. A simple random number was employed to create the sequence order, which was used in this study (10, 35, 20, 40, 25, 15, and 30% MVC). During program execution, the participants were shown both visual and numerical representations of the target forces on the screen. They were instructed to squeeze the force transducer to adjust the cursor toward the specified target levels (refer to Figure 2). Upon reaching each target, the participants released the force on the transducer, causing the cursor to return to the baseline. Subsequently, the system displayed the next target after each release [39]. For delivering visual targets in either a random or sequence order, a number of macros were developed in PowerLab^TM^ ADInstrument 4/35 with LabChart^TM^ (Bella Vista, NSW, Australia). Each block consisted of eight trials, and each trial included seven target forces, which appeared in a sequence order (10, 35, 20, 40, 25, 15, and 30% MVC) or a random order on the computer screen. At the beginning of each experiment, the MVC was individually determined for calibration in each participant, and then two trials were provided as a means of familiarization. After familiarization, two sequences or random blocks with each hand were randomly performed as the baseline measurement. During training, each participant completed eight blocks of the same sequence order with the dominant hand, except for block 6, which was set in a random order. The participants were not aware of the order of sequence during and after the training. Fifteen minutes after the concurrent application of both training and brain stimulation, the participants randomly completed four test blocks as a post-test assessment including sequence right (Seq.R), sequence left (Seq.L), random right (Ran.R), and random left (Ran.L) hand. RTs as the behavioral outcomes were measured in each assessment block.

As shown in Figure 2, RT was the interval between the appearance of a stimulus (force target) on the computer screen and the moment when the force response was taken above a resting range. The mean of the RTs for eight repetitions of the same target force across a block was calculated as the RT for the given target in that block. The ratio RT [(pre-post/pre) × 100] was also measured in each target force for all four test bocks (Seq.R, Seq.L, Ran.R, and Ran.L). 

### 2.3. Transcranial Direct-Current Stimulation (tDCS)

A commercial stimulator (Intelect Advanced Therapy System, Chattanooga, TN, USA) was used to deliver a direct current with an intensity of 0.3 mA [40,41] for 20 min during training. The active electrode (1.5 × 2 = 3 cm^2^) was placed over the left M1, DLPFC, and PPC, and the return electrode (4 × 3 = 12 cm^2^) was placed over the contralateral supraorbital region. The small size of the electrodes yielded a highly focused direct current over the target regions; the current intensity for the small electrode size was adjusted by keeping the current density (0.1 mA/cm^2^) in a safe range. Two electrodes were covered with saline-soaked sponges and strapped in place by two elastic bands [42]. The location of the M1 area was identified using transcranial magnetic stimulation (TMS) and centered on the representational field of the right first interosseous muscle (FDI), which plays a dominant role during SVIPTs [13]. The location of the DLPFC (F3) and the PPC (P3) was determined using the international 10–20 EEG system. To pinpoint the F3 location (the left DLPFC), the process began with identifying the vertex. Next, Fz was located at the front of the vertex by measuring 20% of the total scalp length from the vertex down the mid-sagittal line. F3 was subsequently marked 20% laterally from Fz on the left cortex. In a similar manner as P3 localization at the back of the head, Pz was first found by measuring 20% of the total scalp length from the vertex along the mid-sagittal line towards the back, and then P3’s position was determined, being 20% lateral from Pz. The participants reported the side effects under the electrodes, such as itching, tingling, burning sensations, and burning pain, and adverse effects such as headaches [43]. If the participants reported burning pain or any other side effects such as itching or burning under the electrodes, we injected some normal saline into the sponges using a syringe to keep them wet throughout the experiment [42]. For the sham stimulation group, the same procedure was performed but the current was ramped up to 0.3 mA for 30 s and then ramped down so that the participants felt an initial sensation for 30 s of stimulation. The active electrode was randomly positioned over the three different stimulation areas (M1, DLPFC, or PPC). 

In each experiment (Figure 2), the same procedure was followed: (1) baseline assessment, (2) concurrent training with anodal/sham tDCS, and (3) assessment 15 min after the interventions. The participants randomly performed four blocks consisting of seven trials in either sequential or random orders with either hand (Seq.R, Seq.L, Ran.R, and Ran.L) at two time points: baseline and after intervention.

### 2.4. Data Analysis

Sample size calculation: A power analysis (G-Power v3.1) was carried out for the F test. ANOVA (fixed effects, omnibus, one-way) was used to calculate the sample size for this study. In G-Power, this test can be applied for a non-parametric Kruskal–Wallis test. A total sample size of 48 participants was determined for a power of 0.8, with the alpha set to 0.05 and an effect size of 0.5. 

The normality of the data was assessed using the Kolmogorov–Smirnov (K-S) test. For normally distributed variables, a two-way ANOVA was used with two independent factors (groups and blocks) as the between-subject factors and time (baseline and 15 min after stimulation) as the within-subject factors. A one-way ANOVA was conducted to examine significant differences in the participants’ characteristics among the four groups at the baseline. If normality was violated, a non-parametric analysis, the Kruskal–Wallis test H value, was used to determine differences in the mean rank of the variables among the four groups separate from all the four assessment blocks. If the Kruskal–Wallis test was statistically significant, then pairwise comparisons of group Dunn tests was used to determine differences between each pair of groups. Eta squares (η^2^) were also calculated to determine the effect sizes in this study. An effect size of 0.01 demonstrated a small effect, 0.06 a moderate effect, and 0.14 and above indicated large intervention effects [44]. 

SPSS (version 20) and MATLAB (R2014a) were used to analyze the data in this study. Statistical significance was set to *p* = 0.05.

## 3. Results

The results of the Kolmogorov–Smirnov (K-S) test revealed that the measured temporal variables in our study, i.e., RTs and their ratio of RTs, were not normally distributed. Therefore, the Kruskal–Wallis test was used to determine the differences in these variables for each test block among the four groups. Participant characteristics in terms of age and some other variables such as computer game hours and handedness were normally distributed across the groups; therefore, we used a one-way ANOVA to determine the differences in these characteristics among the four groups.

The results of the one-way ANOVA showed no significant differences in participant characteristics such as age (F = 1.52, *p* = 0.22), MVC (F = 1.46, *p* = 0.24), handedness (F = 0.89, *p* = 0.45), and computer game time (hour in a day) (F = 0.34, *p* = 0.79) among the four groups. All the participants tolerated tDCS and reported no side effects during or after the experimental sessions.

The Kruskal–Wallis test was used to determine the differences in the RTs for each test block among the four groups. As shown in Table 1, there were no significant differences in the mean rank of the RTs for all the target forces among the four groups at the baseline (*p* > 0.05).

### 3.1. Ratio RT for Sequence Blocks in Both Right and Left Hands

**Figure 3:**
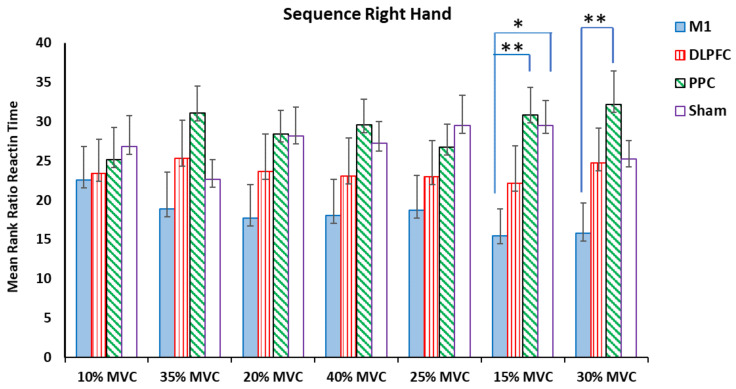
The mean rank of the RT ratio in the sequential right hand block assessment test among four tDCS stimulation sites (M1, DLPFC, PPC, and sham) (* *p* < 0.05, ** *p* < 0.01).

**Figure 4:**
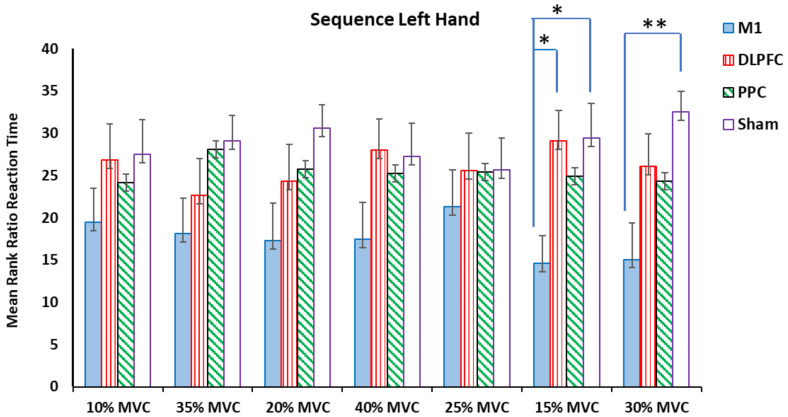
The mean rank ratio RT in the sequence left hand block assessment test among four tDCS stimulation sites (M1, DLPFC, PPC, and sham) (* *p* < 0.05, ** *p* < 0.01).

The results of the Kruskal–Wallis test showed that there were significant differences among the a-tDCS groups in the ratio of RT at target forces of 15% and 30 % MVC for both right and left hands (*p* < 0.05) (Table 2) (Figure 3 and Figure 4). 

For the right trained hand, the results of pairwise comparisons of the groups showed that the participants who had received a-tDCS over the left M1 had a significant elongation in their ratio of RT for a force of 15% MVC compared to the sham (H = −14, *p* = 0.014, η^2^ = 0.29) and M1-PPC (H = −15.33, *p* = 0.007, η^2^ = 0.32). For the force target of 30% MVC, this negative effect was also observed between the M1 and PPC (H = −16.33, *p* = 0.004, η^2^ = 0.34) groups. No significant differences were found in other target forces (Figure 3). 

For the left untrained hand, the Kruskal–Wallis test showed significant differences at the same temporal measured variables, i.e., 15% and 30% MVC (Table 2). The results of pairwise comparisons of the groups showed that M1 compared to the sham (H = −14.8, *p* = 0.009, η^2^ = 0.31) and M1-DLPFC (H = −14.5, *p* = 0.011, η^2^ = 0.3) groups showed an increase in the measured variable for the force target of 15% MVC. Significant differences were found between M1 and sham (H = −17.41, *p* = 0.002, η^2^ = 0.37) in favor of the sham at a force target of 30% MVC (Figure 4). 

No significant differences were found in other target forces (Table 2) (Figure 4). 

### 3.2. Ratio RT for Random Blocks in Both Right and Left Hands

**Figure 5:**
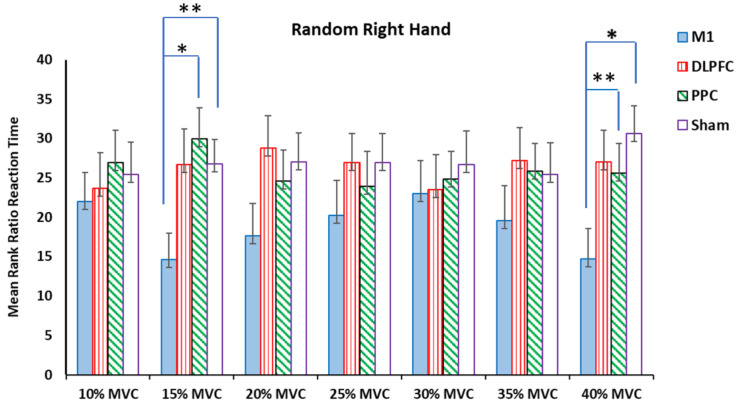
The mean rank ratio of the RT in the random right-hand block assessment test among four tDCS stimulation sites (M1, DLPFC, PPC, and sham) (* *p* < 0.05, ** *p* < 0.01).

For the right trained hand, the results of the Kruskal–Wallis test showed significant differences in the ratio RT for force targets of 15% and 40% of MVC (Table 2). Pairwise comparisons of the groups revealed that a-tDCS on the left M1 had a negative effect on the measured temporal variable at a force target of 15% MVC compared to the sham (H = −12.08, *p* = 0.035, η^2^ = 0.25) and PPC (H = −15.25, *p* = 0.008, η^2^ = 0.31) groups. For the force target of 40% MVC, a-tDCS over the left M1 showed a significant elongation in the ratio of RT compared to the sham (H = −15.8, *p* = 0.005, η^2^ = 0.32) and DLPFC (H = −12.2, *p* = 0.032, η^2^ = 0.25) groups (Figure 5). 

**Figure 6:**
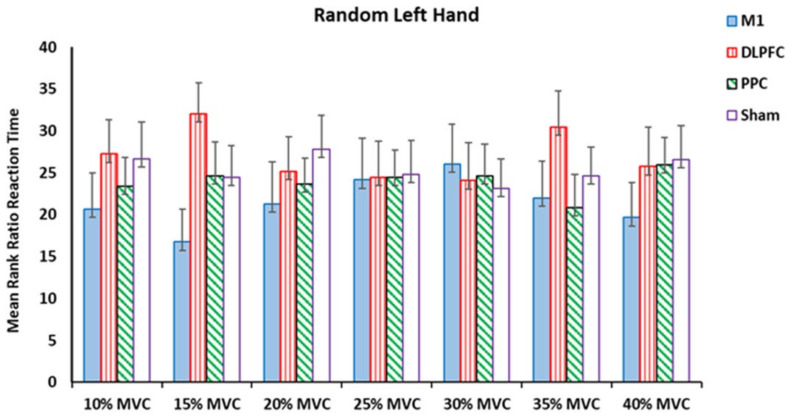
Mean rank ratio RT in the random left-hand block assessment test among four tDCS stimulation sites (M1, DLPFC, PPC, and sham).

For the left untrained hand, no significant effects were found in the ratio of the RT at any force target among the four tDCS stimulation sites (Table 2 and Figure 6). 

Therefore, the results of the non-parametric tests demonstrated that there were some significant differences between the groups in the mean rank of the ratio of the RT for sequence blocks in both the right trained and left untrained hands. For the random blocks, we only found some significant differences between the groups in the right trained hand not in the left hand.

## 4. Discussion

Our findings showed that the participants who received the left M1 stimulation showed a significant increase in the RT ratio for some target forces compared to the sham group, while the DLPFC and PPC stimulations did not modify the RTs within the SVIPT. The observed elongations in the ratio of the RTs after M1-a-tDCS were transferred into the untrained hand in sequence blocks of the SVIPT but not in the random blocks. In the current study, we aimed to assess whether the RTs during an SVIPT was differentially affected by the stimulation of three different areas of the FPC. There were no discernible positive or negative effects detected on the RTs following DLPFC and PPC a-tDCS. However, notable impairments in the RTs were observed subsequent to M1 stimulation.

### 4.1. The Effects of M1 Stimulation on the RTs

We found elongations in the RTs following a single session of M1 stimulation during an SVIPT. Contrary to our results, Waters-Metenier et al. (2014) observed an enhancement in both the execution time and the RT following a 4-day application of bihemispheric M1 a-tDCS with an intensity of 2 mA and an electrode size of 35 cm^2^ during a piano-like key task [45]. They applied multiple sessions of a-tDCS over M1 during motor sequence training. A meta-analysis of tDCS studies revealed that multiple sessions, compared to single session, of a-tDCS over M1 induced significant improvements in behavioral outcome measures in both SRTTs and SVIPTs [46]. This discrepancy can be related to the application of multiple sessions of M1 tDCS. They also stimulated the M1 area with an intensity of 2 mA with a larger electrode size comparable to our study. Likewise, Horvath et al. (2016) found no significant effects of a single session of anodal or cathodal M1 tDCS (2 mA or 1 mA with an electrode size of 35 cm^2^) on a simple motor reaction time task [47]. They suggested that tDCS over M1, regardless of polarity, stimulation intensity, and electrode montage, might not have a positive effect on reaction time in a relatively lower-level motor behavioral task [47]. In the current study, we applied a single session of a-tDCS with an intensity of 0.3 mA and a small electrode size of 3 cm^2^ over M1 during a complex sequential motor task in which participants controlled their force to reach different target forces appearing on the computer screen. Since we aimed to investigate the effects of M1 stimulation on the RTs within the SVIPT, we used a focal small electrode size of 3 cm^2^ to selectively stimulate the M1 area, not nearby areas, such as the primary sensory area, the premotor cortex, or the supplementary motor area. Nitsche et al. (2007) demonstrated that using a smaller stimulation electrode size leads to changes in the excitability of the M1 area that are specific to the muscle representation directly beneath the electrode, without affecting adjacent muscle areas [48]. This was observed by measuring the muscle-evoked potential (MEP) amplitudes in the abductor digiti minim (ADM) and the first dorsal interosseous (FDI) muscles through TMS, following M1 a-tDCS with electrode sizes of 35 cm^2^ and 3.5 cm^2^. When the larger 35 cm^2^ electrode was used, encompassing both muscle representations, a-tDCS similarly affected the MEP amplitudes of both the ADM and FDI muscles. However, with the smaller electrode which only covered the ADM’s representational field, the excitability of the FDI’s cortical representation was not altered. Thus, a smaller electrode on the M1 might activate fewer motor neurons of the muscle representations, leading to a diminished effectiveness of the tDCS stimulation. 

In this study, it is likely that the M1 representations of the muscles involved in the SVIPT task, such as the FDI, were not specifically targeted by the 3 cm^2^ tDCS electrode. Moreover, the use of a small electrode size potentially limits the connectivity-driven effects of tDCS on distant brain regions. Boros et al. (2008) found that the anodal stimulation of the premotor (0.1 mA, 3.5 cm^2^, 13 min) modifies the intracortical excitability of the ipsilateral M1 [49]. Therefore, the activity modulation of adjacent interconnected areas such as the premotor might impact RT processing within SVIPTs. Elbert and co-workers observed that the application of anodal tDCS (0.26 mA, 1.5 cm^2^) at the vertex close to the supplementary motor area can improve RTs in a tone–noise sequence task [50]. The stimulation of adjacent areas to the M1 such as the premotor or supplementary motor areas on RT processing within SVIPTs should be explored in future studies.

### 4.2. The Effects of DLPFC and PPC Stimulation on the RTs

While the involvement of the DLPFC has been shown in neuroimaging studies in the early stage of motor learning [51,52,53], in our study, we found no significant effects of left DLPFC stimulation on the RTs within the SVIPT compared to the sham stimulation. Marshall et al. (2005) showed that both anodal and cathodal stimulation (260 μA; 15 s on/15 s off; 8 mm diameter; 15 min) impaired reaction time processing in a working memory task [54]. They used tDCS with a very low amplitude of 260 μA intermittently during a working memory task, which was different from the SVIPT used in the current study. We stimulated left DLPFC with an intensity of 0.3 mA in a constant, not intermittent, manner during a pinch force sequential task. In contrast, enhancement effects in stop signal reaction time were observed following right DLPFC a-tDCS stimulation (0.5 mA, 9 cm^2^, 19 min) with extra cephalically montage on the contralateral deltoid [55]. In the aforementioned study, they applied a-tDCS over the right DLPFC and observed improvements in cognitive inhibition processes in stop signal reaction time by making fewer omission errors [55]. Contrary to their results, we observed no positive effects on the RTs within the SVIPT in the participants who had received left DLPFC a-tDCS with a contra-orbital montage. They also found that the increase in skill was greater following right DLPFC stimulation than left DLPFC stimulation. With regard to the positive effects observed in the RTs following right DLPFC stimulation in a recognition reaction time task, it might be valuable in future studies to explore the effects of right DLPFC tDCS on the RTs within an SVIPT. 

In the current study, we also observed no significant effects of left PPC stimulation on the RTs within the SVIPT. However, the relevance of the left PPC as an anticipatory center for precise sensorimotor timing has been identified in a study by Krause et al. (2012). They applied 1 Hz repetitive transcranial magnetic stimulation (rTMS) over the left PPC, right PPC, and visual cortex of healthy individuals for 10 min and showed that activity in the left PPC was essential for the precise execution of sensorimotor tasks, especially when the quick adjustment of movements is required in response to external stimuli [56]. No positive effects of a-tDCS stimulation over the left PPC were observed in our study. Contrary to our results, Heinen et al. (2016) have shown that bilateral PPC stimulation, independent of electrode configuration, can enhance visual working memory precision [57]. They also found that cathodal but not anodal tDCS over the right PPC can improve general working memory precision [57]. These discrepancies can be explained by the different methodologies used in these studies. We applied unilateral PPC stimulation with a low intensity and a small electrode size during an SVIPT, while they applied bilateral PPC stimulation with an intensity of 1.5 mA and an electrode size of (6.5 × 4.5 cm) during a visual working memory task. Although the SVIPT task used in the current study was not similar to theirs, bilateral PPC stimulation or cathodal PPC stimulation within SVIPTs should be explored in future studies. 

### 4.3. The Effects of Stimulation on Transfer Learning

In this study, we also aimed to assess the differential effects of brain stimulation over three different areas of the FPC on the transfer of learning within an SVIP. No transfer learning was observed in the DLPFC and PPC stimulation groups. We also observed that the impairments in the ratio of the RTs in the M1 group were transferred to the left untrained hand. The present result is in line with a study by Keitel et al. (2018) showing that a-tDCS applied to the right M1 impairs implicit motor sequence learning of both hands [58]. They applied a-tDCS (9 cm^2^, 0.25 mA, 10 min) over the right (ipsilateral) M1 during an SRTT with the right trained hand [58]. In the current study, we applied a-tDCS over the left (contralateral) M1 during SVIPT training with the right hand. In both studies, the participants were not aware of the underlying sequential pattern, indicating implicit learning, which is primarily mediated by the cortico–striatal–cerebellar network [59]. A PET study showed that an improvement in reaction time during implicit sequence learning is associated with increased activity in the contralateral primary sensorimotor cortex (SM1), while increased activity of the FPN network is observed during explicit sequence learning, when the participants are aware of the sequence [60]. The negative intermanual transfer in the M1 group, in the current study, showed that there is an interaction between the bilateral M1 in implicit sequence learning, which support the hypothesis of interhemispheric connections and can transfer impairments of RT measurements. It is well-known that the corpus callosum is the main neural pathway that connects left and right cortical areas, including the prefrontal, motor, somatosensory, parietal, and occipital areas on either hemisphere, and enables the transfer of motor skills from one hand to the other hand [61]. Bilateral M1 activation has been reported when participants performed SRTT training with one hand [62,63], which reflects how these interhemispheric connections work during training. Therefore, training with one hand led to excitatory or inhibitory activity in both hemispheres [61,62]. Our finding suggested that, through interhemispheric connections, not only improvement but also the attenuation of performance with one hand can be transferred to the opposite hand. Considering the fact that the a-tDCS technique used in this study showed no significant improvement on the RTs, further research is needed to investigate the impact of different stimuli conditions of tDCS in terms of electrode montage, current intensity, or electrode size on the RTs in SVIPTs. 

## 5. Limitations

The findings in the current study should be interpreted in light of a number of limitations. We included healthy young individual participants, so we cannot generalize our findings to the elderly population or patients with neurological disorders. We computed the sample size required for a parametric test in G-Power in this study. For the non-parametric test, we needed to add at least 15% to the total sample size. Therefore, the recruitment of more participants could increase the power of this study to find significant differences between the groups, if any exist. This study was single-blinded, meaning that participants were not aware of the type of stimulation they received while the researcher was not blinded to the intervention groups, which may increase the risk of bias. We used a single session of brain stimulation over the three areas of the FPC; the application of multiple sessions should be investigated in future studies. We did not have a control group to assess the specificity of the training effects on the measured variables, which is another limitation of our study. Long-term outcome measures were not evaluated in this study. Therefore, it is suggested that future studies investigate the effects of brain stimulation on behavior outcomes at longer follow-up times within SVIPTs. The application of tDCS with different stimulation parameters such as amplitude, electrode size, and stimulation sites during implicit or explicit sequence learning in a task such as SVIPT should be explored in future studies.

## 6. Conclusions

Our results demonstrated an elongation in the ratio of the RTs following a single session of left M1 stimulation compared to the sham group. No significant effects were observed after left DLPFC and PPC stimulation on the ratio of the RTs in implicit sequence learning during an SVIPT compared to the sham group’s stimulation. Our study found that stimulating the left M1 area led to a slight increase in the reaction times (RTs) in some target forces compared to the sham group, but the stimulation of the left DLPFC and PPC did not affect the RTs during implicit sequence learning tasks. Overall, using a-tDCS on the M1, the DLPFC, or the PPC at 0.3 mA did not significantly enhance the RTs in the task we studied. These results suggest that targeting other brain regions, like the premotor cortex, supplementary motor areas, or the cerebellum, might offer more promise in reducing the RTs for implicit motor sequence learning. Interestingly, the increase in the RTs seen with M1 stimulation was also noted in the non-trained hand but only for certain sequences. This indicates a need for further research to identify the most effective tDCS targets for improving the RTs and learning transfer across hands in similar tasks. 

## Figures and Tables

**Table 1:** The results of the Kruskal–Wallis test for the four test blocks on the mean rank of ratio RT (either sequential or random blocks with either hand) among the four stimulation groups (M1, DLPFC, PPC, and sham). RT: reaction time; Seq: sequence; Ran: random; R: right; and L: left.

Baseline RT	Block		Group				
		M1	DLPFC	PPC	Sham	χ^2^	*p*
10% MVC	Seq.R	20.42	25.88	24.17	27.54	1.710	0.635
	Seq.L	18.79	30.63	21.46	27.13	5.281	0.152
	Ran.R	24.13	22.46	25.17	26.25	0.479	0.924
	Ran.L	20.46	28.50	23.46	25.58	2.118	0.548
15% MVC	Seq.R	20.50	21.54	29.17	26.79	3.170	0.366
	Seq.L	18.67	26.92	26.00	26.42	2.804	0.423
	Ran.R	15.92	26.50	28.79	26.79	6.206	0.102
	Ran.L	17.88	28.75	25.54	25.83	3.969	0.265
20% MVC	Seq.R	19.83	22.83	27.50	27.83	2.735	0.434
	Seq.L	19.08	24.92	26.92	27.08	2.573	0.462
	Ran.R	20.42	28.42	22.00	27.17	2.778	0.427
	Ran.L	23.33	25.08	23.88	25.71	0.217	0.975
25% MVC	Seq.R	23.63	24.38	25.21	24.79	0.084	0.994
	Seq.L	23.25	24.92	25.42	24.42	0.158	0.984
	Ran.R	19.58	27.58	22.83	28.00	2.982	0.394
	Ran.L	24.42	23.50	26.08	24.00	0.230	0.973
30% MVC	Seq.R	17.58	24.54	28.38	27.5	4.400	0.221
	Seq.L	16.58	27.13	24.75	29.54	5.819	0.121
	Ran.R	23.50	25.88	23.83	24.79	0.209	0.976
	Ran.L	24.92	22.25	24.92	25.92	0.454	0.929
35% MVC	Seq.R	18.71	23.92	30.17	25.21	4.072	0.254
	Seq.L	19.54	25.50	26.13	26.83	2.062	0.560
	Ran.R	20.96	25.50	23.38	28.17	1.730	0.630
	Ran.L	22.29	24.79	25.46	25.46	0.416	0.937
40% MVC	Seq.R	21.42	22.67	27.88	26.04	1.631	0.652
	Seq.L	19.25	27.13	25.17	26.46	2.372	0.499
	Ran.R	17.00	28.04	24.58	28.38	5.132	0.162
	Ran.L	22.08	22.54	28.04	25.33	1.403	0.705

**Table 2:** The results of the Kruskal–Wallis test on the mean rank of ratio RT in the four assessment blocks (either sequential or random blocks with either hand) among the four stimulation groups (M1, DLPFC, PPC, and sham) (* *p* < 0.05).

Ratio RT (Pre-Post/Pre) × 100	Sequence Block		Random Block	
	Right Hand (Seq.R)	Left Hand (Seq.L)	Right Hand (Ran.R)	Left Hand (Ran.L)
10% MVC	H (3) = 0.65, *p* = 0.883 η^2^ = 0.053	H (3) = 2.42, *p* = 0.49 η^2^ = 0.013	H (3) = 0.834, *p* = 0.841 η^2^ = 0.049	H (3) = 1.72, *p* = 0.632 η^2^ = 0.029
15% MVC	H (3) = 9.27, *p* = 0.026 * η^2^ = 0.143	H (3) = 8.79, *p* = 0.032 * η^2^ = 0.132	H (3) = 8.31, *p* = 0.04 * η^2^ = 0.121	H (3) = 7.2, *p* = 0.066 η^2^ = 0.095
20% MVC	H (3) = 4.59, *p* = 0.204 η^2^ = 0.036	H (3) = 5.5, *p* = 0.138 η^2^ = 0.057	H (3) = 4.34, *p* = 0.226 η^2^ = 0.03	H (3) = 1.36, *p* = 0.714 η^2^ = 0.037
25% MVC	H (3) = 4.01, *p* = 0.261 η^2^ = 0.023	H (3) = 0.821, *p* = 0.845 η^2^ = 0.05	H (3) = 1.84, *p* = 0.606 η^2^ = 0.026	H (3) = 0.014, *p* = 1.000 η^2^ = 0.068
30% MVC	H (3) = 8.23, *p* = 0.041 * η^2^ = 0.119	H (3) = 9.5, *p* = 0.023 * η^2^ = 0.148	H (3) = 0.49, *p* = 0.92 η^2^ = 0.057	H (3) = 0.275, *p* = 0.965 η^2^ = 0.062
35% MVC	H (3) = 4.81, *p* = 0.186 η^2^ = 0.041	H (3) = 4.73, *p* = 0.192 η^2^ = 0.039	H (3) = 2.07, *p* = 0.55 η^2^ = 0.021	H (3) = 3.36, *p* = 0.339 η^2^ = 0.008
40% MVC	H (3) = 4.68, *p* = 0.196 η^2^ = 0.038	H (3) = 4.24, *p* = 0.236 η^2^ = 0.028	H (3) = 8.57, *p* = 0.035 * η^2^ = 0.127	H (3) = 1.92, *p* = 0.587 η^2^ = 0.025

## Data Availability

The raw data supporting the conclusions of this article will be made available by the authors on request.
